# Role of GLIS3 in thyroid development and in the regulation of gene expression in thyroid specific Glis3KO mice

**DOI:** 10.21203/rs.3.rs-3044388/v1

**Published:** 2023-07-07

**Authors:** Hong Soon Kang, Sara A Grimm, Xiao-Hui Liao, Anton M. Jetten

**Affiliations:** National Institutes of Health; National Institutes of Health; The University of Chicago; NIEHS, NIH

**Keywords:** GLIS3, thyroid development, thyroid follicular cell proliferation, thyroid hormone biosynthesis, hypothyroidism, TSH signaling

## Abstract

Loss of GLI-Similar 3 (GLIS3) function in mice and humans causes congenital hypothyroidism (CH). In this study, we demonstrate that GLIS3 protein is first detectable at E15.5 of murine thyroid development, a time when GLIS3 target genes, such as *Slc5a5 (Nis),* become also expressed. We further show that *Glis3KO* mice do not display any major changes in prenatal thyroid gland morphology indicating that CH in *Glis3*KO mice is due to dyshormonogenesis rather than thyroid dysgenesis. Analysis of thyroid-specific *Glis3* knockout (*Glis3*-Pax8Cre) mice fed either a normal or low-iodine diet (ND or LID) revealed that, in contrast to ubiquitous *Glis3KO* mice, thyroid follicular cell proliferation and the expression of cell cycle genes were not repressed suggesting that the inhibition of thyroid follicular cell proliferation in ubiquitous *Glis3*KO mice is related to loss of GLIS3 function in other cell types. However, the expression of several thyroid hormone biosynthesis-, extracellular matrix (ECM)-, and inflammation-related genes was still suppressed in *Glis3*-Pax8Cre mice particularly under conditions of high blood levels of thyroid stimulating hormone (TSH). We further demonstrate that treatment with TSH, protein kinase A (PKA) or adenylyl cyclase activators or expression of constitutively active PKA enhances GLIS3 protein and activity, suggesting that GLIS3 transcriptional activity is regulated in part by TSH/TSHR-mediated activation of the PKA pathway. This mechanism of regulation provides an explanation for the dramatic increase in GLIS3 protein expression and the subsequent induction of GLIS3 target genes, including several thyroid hormone biosynthetic genes, in thyroid follicular cells of mice fed a LID.

## Introduction

CH is one of the most common neonatal endocrine disorders that has been subclassified into thyroid dysgenesis caused by abnormal thyroid development, and dyshormonogenesis caused by defects in thyroid hormone (TH; T3 and T4) biosynthesis [1–6]. Abnormal thyroid gland organogenesis accounts for 80–85% of patients with primary CH. During embryonic thyroid development foregut endoderm cells give rise to thyroid progenitors, which subsequently differentiate along the follicular cell lineage leading to the formation of the TH-producing thyroid follicles [7, 8]. Several transcription factors (TFs), including paired box 8 (PAX8), NK2 homeobox 1 (NKX2.1; TTF1), NKX2.5, forkhead box E1 (FOXE1; TTF2), and hematopoietically expressed homeobox (HHEX), play a critical role in the regulation of thyroid gland development [7]. Loss-of-function mutations in these genes cause thyroid dysgenesis or athyreosis [3, 6, 9, 10]. Mutations in genes critical for TH biosynthesis, including sodium iodide symporter (*NIS; SLC5A5*), pendrin (*PDS; SLC26A4*), thyroglobulin (*TG*), thyroperoxidase (*TPO*), thyroid stimulating hormone receptor (*TSHR*), and dual oxidase 2 (*DUOX2*), have been causally linked to thyroid dyshormonogenesis [11–13].

Krüppel-like zinc finger protein, GLI-Similar 3 (GLIS3), was recently identified as a critical regulator of thyroid follicular cell functions [14, 15]. Loss-of-function mutations in human *GLIS3* cause a syndrome characterized by neonatal diabetes and congenital hypothyroidism (NDH) [16–24], while single nucleotide polymorphisms in *GLIS3* are associated with thyroid dysfunction and increased risk of CH [25–32]. Ubiquitous Glis3 knockout (*Glis3KO*) mice exhibit a very similar phenotype as human NDH patients, including the development of neonatal diabetes and CH [33, 34]. TH biosynthesis in thyroid follicular cells is greatly suppressed in postnatal *Glis3*KO mice [15, 33]. The development of CH in *Glis3*KO is at least in part related to dyshormonogenesis due to the transcriptional repression of several TH biosynthesis-related genes [33]. However, whether GLIS3 also plays a role in the regulation of murine thyroid gland development has not yet been clearly established. In this study, we provide evidence indicating that GLIS3 does not play a major role in mouse embryonic thyroid morphogenesis suggesting that the development of CH in *Glis3*KO mice is due to dyshormonogenesis rather than thyroid dysgenesis.

In this study, we further demonstrate that in contrast to ubiquitous *Glis3*KO mice, proliferation of thyroid follicular cells was not inhibited in thyroid-selective *Glis3* knockout mice fed a LID (*Glis*3-Pax8Cre(LID)), whereas the expression of TH biosynthesis- and extracellular matrix (ECM)-related genes was still repressed. These observations indicate that the inhibition of proliferation and cell cycle-related genes in ubiquitous *Glis3*KO thyroid is related to changes in gene expression in other GLIS3 target tissues that indirectly affect thyroid follicular cell proliferation. Studies showing that GLIS3 is particularly critical for the transcriptional regulation of several genes induced by TSH [14, 15, 35], raised the question whether GLIS3 activity itself is controlled by TSH signaling. This hypothesis is supported by data showing that GLIS3 protein is greatly induced in thyroid follicular cells of mice fed a LID and that the level of GLIS3 protein and its transcriptional activity is significantly enhanced in PCCl3 cells by TSH, cAMP or the expression of constitutively active PKA. These findings are consistent with the hypothesis that GLIS3 protein level and transcriptional activity are controlled by the TSH-TSHR signaling pathway, at least in part through the activation of PKA. This mechanism would explain the dramatic induction of GLIS3 target genes, including several thyroid hormone biosynthetic genes, in thyroid follicular cells when TSH levels are highly elevated, such as in LID conditions. Targeted expression or regulation of GLIS3 in thyroid follicular cells may provide new therapeutic strategies to manage certain subtypes of CH.

## Materials and methods

### Mice and diet

*Glis3*-EGFP mice (C57BL/6-Glis3 < tm3(Glis3-EGFP)Amj>) expressing a GLIS3-EGFP fusion protein, and the ubiquitous *Glis3*-deficient *Glis3*KO1 (B6.Glis3 < tm1AmJ>) and *Glis3*KO2 (C57BL/6-Glis3 < m3(mCherry)Amj>) mice were described previously [33, 36–38]. Conditional *Glis3* knockout mice, *Glis3*-Pax8Cre (CKO), in which the expression of Cre causes a cell type-specific deletion of exon 5 encoding zinc finger 3 and 4 of GLIS3, were generated by crossing Glis3^fl/fl^ mice [39] with Pax8Cre mice (B6.129P2(Cg)-Pax8^tm1.1(cre)Mbu^/J; Jackson Laboratory # 028196). Mice were routinely fed an NIH-31 diet (ND; Harlan, Madison, WI) or fed a low-iodine diet (LID; TD95125 diet, Harlan) for 6 days.

### Histology and immunohistochemistry

For histological analysis, thyroid glands from different stages of mouse embryonic development and from postnatal mice were fixed overnight in 4% paraformaldehyde, washed with PBS, and embedded in paraffin. Sections (5 μm) were then stained with hematoxylin and eosin (H&E). For immunohistochemical analysis, fixed thyroid glands were washed, transferred into 30% sucrose for 2–3 days, and then frozen in OCT compound (Tissue-Tek). Frozen sections (10 μm) were obtained using a cryostat (Leica Biosystems, Deer Park, IL) and subsequently immunostained with either a GFP (A-11122, Life Technology or GFP-1010, Aves Labs), PAX8 (NBP1-32440, Novus Biologicals), NKX2.1 (ab76013, Abcam), CDH16 (PA5-57589, ThermoFisher), ZO-1 (61-7300, ThermoFisher), E-Cadherin (CDH1; U3254, Sigma-Aldrich), PECAM1 (#550274, BD Pharmingen, San Diego, CA), pRPS6 (#2211, Cell Signaling), pan collagen (PA1-36058, ThermoFisher) or SLC5A5 (gift from Dr. N. Carrasco) antibody and then incubated with Alexa Fluor 488- or 594-conjugated secondary antibodies (ThermoFisher) for 1 h at room temperature. Wheat germ agglutinin (WGA) conjugated with Alexa Fluor 647 was used to stain cell membrane and nuclei with DAPI Prolong Diamond (both from ThermoFisher). Proliferation of thyroid follicular cells were analyzed by Click-iT EdU assay kit (ThermoFisher) as described previously [33]. Fluorescence was examined with a Zeiss LSM780 confocal microscope. Metamorph Premier Offline version 7.10.3.279 (Molecular Devices LLC, Sunnyvale, CA) was used to quantify Images of GLIS3 and PAX8 positive cells. A median filter was applied to the red and green channels and Multi Wavelength Cell Scoring was used to count the total number of red cells and double positive cells.

### Measurement of serum and tissue TSH and TH levels

Serum T3, T4, and TSH levels were measured by radioimmunoassay as described in detail previously [33, 40].

### RNA-Seq analysis

Thyroid glands from 4-week-old WT (n = 4) and *Glis3*-Pax8Cre (n = 4) mice fed an ND or a LID for 6 days were collected and RNAs isolated with a RNAqueous-Micro total RNA isolation kit (ThermoFisher). mRNA isolation and library generation were carried out with a TruSeq Stranded mRNA and TruSeq RNA Library preparation kits (Illumina Inc., San Diego, CA), respectively. Sequence was read by paired-end sequencing using a NextSeq500 or NovaSeq 6000 Sequencing System (Illumina). Raw reads pairs were filtered to retain only those with average base quality score > 20 for both read ends. Filtered read pairs were mapped to the mm10 reference assembly via STAR v2.5 [41] with parameters “--outSAMattrIHstart 0 --outFilterType BySJout --alignSJoverhangMin 8 --limitBAMsortRAM 55000000000 --outSAMstrandField intronMotif --outFilterIntronMotifs RemoveNoncanonical”. Counts per gene were extracted via featureCounts (Subread v1.5.0-p1) [42] with parameters “-s0 -Sfr -p” for RefSeq gene models as downloaded from the UCSC Table Browser on November 7 2017. Differential gene expression analysis was performed using DESeq2 v1.14.1 [43]. For the purposes of pathway analysis, differentially expressed genes are defined at FDR threshold 0.01 and fold change > 2 or < −2.

### QRT-PCR analysis

The expression of mRNA from thyroid gland was examined by QRT-PCR analysis using TaqMan and SYBR system. RNA from thyroid glands was extracted using RNAqueous-Micro total RNA isolation kit and reverse-transcribed using the high-capacity cDNA reverse transcription kit (both from ThermoFisher). QRT-PCR reaction in triplicate for each sample was carried out using StepOnePlus Real-time PCR system (Applied Biosystem). Primer sequences are listed in Supplementary Table 1.

### Plasmids and reagents

Expression plasmid encoding constitutively active PKA (PKA catalytic subunit Calpha) was obtained from Addgene (#15310). p3xGLISBS-Luc and p3XFlag-Glis3 were described previously [33, 44]. Forskolin, FK506, and 8-Bromo-cAMP (8BrcAMP) were obtained from Millipore Sigma (Burlington, MA), H89 and Gö6976 from BioVision (Waltham, MA), and Trametinib from Cayman Chemical Company (Ann Arbor, MI)

### Cell culture and reporter assay

Rat thyrocyte PCCl3 cells were cultured in F12 Media supplemented 5% FBS with 1 mIU/ml TSH, 10 μg/ml Insulin, 10 ng/ml somatostatin, 10 ng/ml 1-glycyl-histidyl-lysine, 5 μg/ml apo-transferrin, and 10 nM hydrocortisone. To examine GLIS3 transcription activity, PCCl3 cells were plated, grown for 3–4 days in the absence of TSH, and then co-transfected with pCMV-βGal (control), p3XFlag-Glis3, and the luciferase reporter p3xGLISBS-Luc. After 24 h, cells were treated with TSH or with the compound indicated and 24 h later assayed for luciferase and β-galactosidase activity with a Luciferase assay system (Promega) and Luminescent β-gal detection kit (Takara), respectively. Luciferase activity was normalized to β-gal activity. Assays were performed in triplicate.

### Western blot analysis

PCCl3-pIND20-Glis3 cells, expressing a doxycycline (Dox)-inducible GLIS3 tagged with Flag and HA at the N- and C-terminus, respectively, were generated by infection of pIND20-Flag-Glis3-HA lentivirus [45]. To evaluate the expression of GLIS3, PCCl3-pIND20-Glis3 cells were treated with 100 ng/ml Dox for 72–96 h in the absence of TSH and subsequently with TSH for 24 h or as indicated. Nuclear extracts were prepared as described [46] and GLIS3-HA examined by Western blot analysis with an HA antibody (#3724, Cell Signaling). β-actin (MA5-15739, ThermoFisher) was used as internal control.

### Statistical analysis

P values were calculated by one-way ANOVA.

## Results

### Expression of GLIS3 during thyroid folliculogenesis

**Figure 1:**
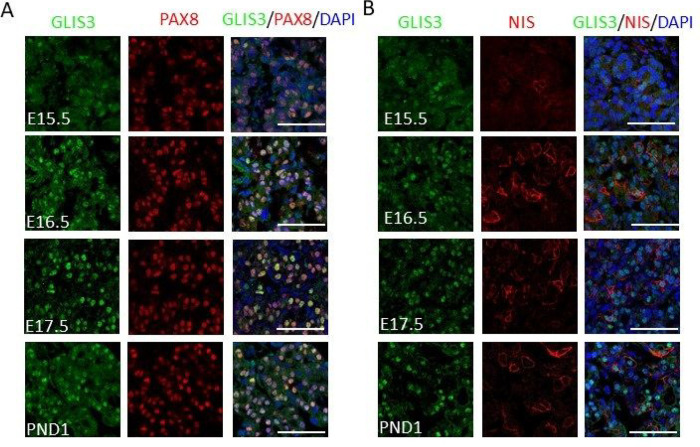
GLIS3 protein is first detectable during E15.5 of mouse embryonic thyroid gland development. **A, B** GLIS3 expression was examined in thyroid glands from GLIS3-EGFP mice at E15.5, E16.5, E17.5 and PND1 by immunofluorescent staining using antibodies for GFP (GLIS3, green) and PAX8 (red) (**A**), and for GFP (GLIS3, green) and NIS (red) (**B**). Nuclei were stained with DAPI. Scale bars: 50 mm.

Previously, we reported that GLIS3 protein is expressed in postnatal murine thyroid follicular cells and that it functions as a critical transcriptional regulator for several genes required for thyroid hormone biosynthesis [33]; however, whether GLIS3 plays a role in the regulation of mouse thyroid organogenesis, has not been clearly established. To study this, we monitored the expression of GLIS3 protein in *Glis3*-EGFP mice at different stages of embryonic development in comparison to PAX8, which is critical for early thyroid organogenesis, and NIS, a marker of differentiated thyroid follicular cells [4]. In contrast to the PAX8 protein, GLIS3 protein was undetectable in the thyroid gland at embryonic day 13.5 (E13.5) (Supplementary Fig. 1). Weak expression of GLIS3 protein was first observed at E15.5 in the nucleus of PAX8^+^ cells (Fig. 1A) and the intensity of GLIS3 immunostaining was significantly increased at E16.5. Similarly, expression of NIS protein, encoded by the GLIS3 target gene *Slc5a5*, was also first observed at E15.5–16.5 consistent with previous reports (Fig. 1B)[7, 47]. GLIS3 protein remained restricted to thyroid follicular cells throughout embryonic and early postnatal thyroid development (Fig. 1A).

**Figure 2:**
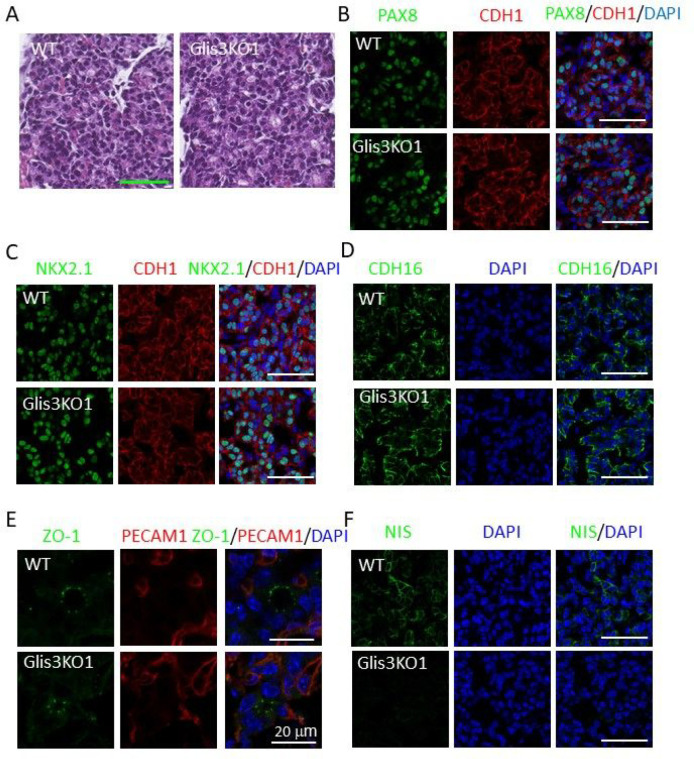
Loss of GLIS3 function does not cause major changes in mouse thyroid gland development. **A** H&E-stained sections of thyroid glands from E17.5 WT and *Glis3*KO1 mouse embryos. **B– F** Representative images of sections of thyroid glands from E17.5 WT and *Glis3*KO1 embryos immunostained with antibodies against PAX8 and CDH1 (**B**), NKX2.1 and CDH1 (**C**), CDH16 (**D**), ZO-1 and PECAM1 (**E**), and NIS (**F**). PECAM1 was used to stain endothelial cells. Nuclei were stained with DAPI. All scale bars are 50 mm, except for 20 mm in E.

Next, we examined whether loss of GLIS3 function had any major effect on thyroid gland morphology and folliculogenesis in *Glis3*KO1, in which the coding sequence of the 5th zinc finger was deleted [36], and *Glis3*KO2, in which the mCherry coding sequence with stop codon was inserted into exon 3 [33, 37]. H&E histochemical staining of sections of E17.5 WT and *Glis3*KO1 thyroid glands revealed no significant differences in thyroid gland morphology (Fig. 2A) and showed a comparable pattern of PAX8 and NKX2.1 immunostaining (Fig. 2B and C). Similar observations were made in thyroid glands from postnatal day 3 (PND3) *Glis3*KO2 mice (Supplementary Fig. 2A–C). Immunostaining further demonstrated that the basolateral localization of the polarity markers, CDH1 and CDH16, and the localization of ZO-1 to apical tight junctions [48–50] remained unchanged suggesting that loss of GLIS3 function did not significantly alter thyroid follicular cell polarity (Fig. 2B–E, Supplementary Fig. 2B-E). In contrast, the expression of NIS protein was greatly reduced in thyroid follicular cells of E17.5 *Glis3*KO1 (Fig. 2F) and PND3 *Glis3*KO2 mice (Supplementary Fig. 2F). Together, these observations indicate that GLIS3 does not play a major role in the regulation of thyroid organogenesis and thyroid folliculogenesis during mouse embryonic development. This is consistent with the hypothesis that in GLIS3-deficient mice the development of congenital hypothyroidism is due to dyshormonogenesis rather than thyroid dysgenesis [33].

### Analysis of Glis3 -Pax8Cre thyroid gland phenotype

**Figure 3:**
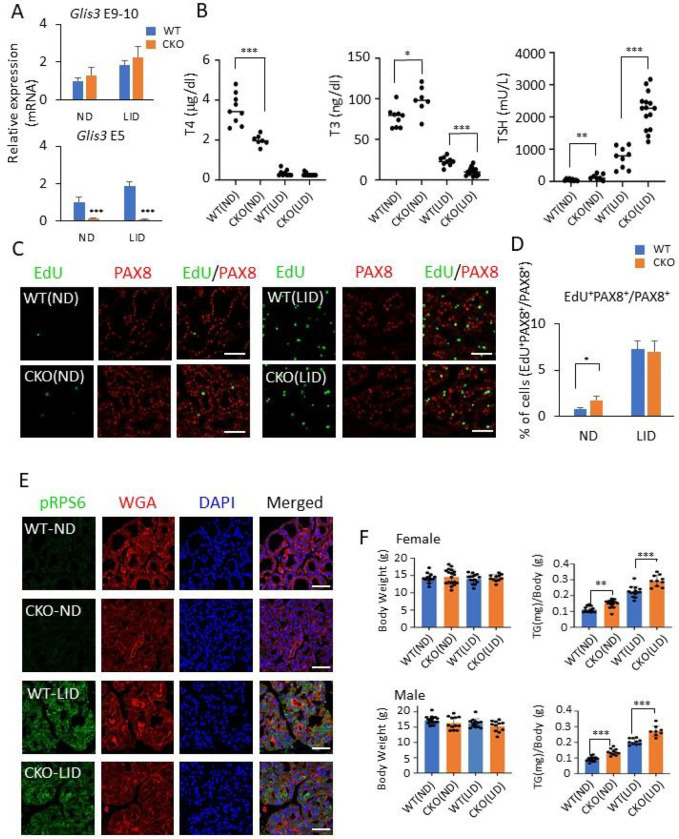
Loss of GLIS3 function does not suppress thyroid follicular cell proliferation in *Glis3*-Pax8Cre(LID) mice. **A** QRT-PCR analysis of Glis3 mRNA expression confirming the specific deletion of exon 5 (E5) in the thyroid gland of *Glis3*-Pax8Cre mice (CKO). Exon 9–10 (E9–10), a non-deleted region of *Glis3*, served as control. **B** Serum levels of T3, T4, and TSH in 5-week-old WT(ND), *Glis3*-Pax8Cre(ND) (referred to as CKO(ND)), WT(LID), and *Glis3*-Pax8Cre(LID) (referred to as CKO(LID)). * p<0.05, ** p<0.01, *** p<0.001. **C** Cell proliferation in thyroid glands from WT and *Glis3*-Pax8Cre mice fed a ND or LID was analyzed by EdU incorporation (green) as described in Material and Methods. Thyroid follicular cells were stained with a PAX8 antibody (red). In contrast to ubiquitous Glis3-knockout mice [33], thyroid follicular cell proliferation was not suppressed in *Glis3*-Pax8Cre(LID) mice. Scale bars: 50 mm. **D** The percentages of the number of PAX8^+^ cells that stained EdU^+^ were calculated and plotted. **E** Activation of the mTOR pathway, as indicated by pRPS6 immunofluorescent staining, was not suppressed in thyroids from *Glis3*-Pax8Cre(LID) mice. pRBS6 (green), WGA (red), DAPI (blue). Scale bars: 50 mm. ** p<0.01, ** p<0.01, *** p<0.001. **F** The relative weight of thyroid glands (TG) in male and female WT(LID) and *Glis3*-Pax8Cre(LID) were increased compared to mice fed a ND, while total body weights were not changed.

In addition to hypothyroidism, ubiquitous *Glis3* knockout mice exhibit other abnormalities, including severe hyperglycemia and hypoinsulinemia, due to defects in pancreatic β cell development and insulin production [15, 38, 39]. Insulin-like growth factors (IGFs) and insulin have been reported to play a critical role in the regulation of thyroid follicular cell proliferation and thyroid gene expression, including *Slc5a5* [51–57]. To investigate whether changes related to other tissues, such as hypoinsulinemia, contributed to the *Glis3*KO thyroid phenotype, we analyzed the thyroid gland phenotype in thyroid-selective *Glis3* knockout mice, *Glis3*-Pax8Cre (referred as conditional knockout or CKO in the Figures), in which Pax8Cre efficiently (> 90%) deleted exon 5 in *Glis3* in the thyroid gland, but not in the pancreas (Fig. 3A and Supplementary Fig. 3A). We demonstrated that in contrast to ubiquitous *Glis3*KO mice, pancreatic insulin expression and non-fasting blood glucose levels were not changed in *Glis3*-Pax8Cre mice (Supplementary Fig. 3B, C) confirming that these mice did not develop hypoinsulinemia/hyperglycemia [58]. However, serum T4 levels were still significantly decreased (44%) in *Glis3*-Pax8Cre(ND) mice, while levels of serum TSH and T3 were slightly elevated (Fig. 3B). Serum T4 and T3 levels were greatly decreased in both WT(LID) and *Glis3*-Pax8Cre(LID) mice fed a LID, whereas TSH was greatly increased but to a significantly greater extent in *Glis3*-Pax8Cre(LID) mice (Fig. 3B).

It is well established that elevated TSH levels, as under LID conditions, cause a dramatic increase in thyroid follicular cell proliferation [54, 57, 59]. We previously reported [33] that in contrast to WT(LID) mice, thyroid follicular cell proliferation and mTOR activation was not increased in ubiquitous *Glis3*KO2(LID) mice and that thyroid gland hypertrophy was not observed. In contrast, *Glis3*-Pax8Cre(LID) mice did develop thyroid gland hypertrophy and the percentage of EdU^+^PAX8^+^ cells was similar to that in WT(LID) (Fig. 3C, D) indicating that thyroid follicular cell proliferation was not repressed in *Glis3*-Pax8Cre(LID) mice. This is consistent with data showing that mTOR activation (pRSP6 staining), a major signaling pathway driving TSH-induced proliferation of thyroid follicular cells, was increased to a similar extent in both *Glis3*-Pax8Cre(LID) and WT(LID) thyroid glands (Fig. 3E). This increase in thyroid follicular cell proliferation correlated with the development of thyroid gland hypertrophy in both male and female *Glis3*-Pax8Cre(LID) mice (Fig. 3F). In fact, thyroid hypertrophy was more pronounced in *Glis3*-Pax8Cre(LID) than in WT(LID) mice as indicated by the larger increase in thyroid weight (Fig. 3F). No significant difference in total body weights was observed between *Glis3*-Pax8Cre and WT mice (Fig. 3F). Together, these results suggested that GLIS3 does not play a direct role in the regulation of TSH-stimulated thyroid follicular cell proliferation and that the repression of cell proliferation in ubiquitous *Glis3*KO(LID) mice appears to be related to changes in other cell types that indirectly affect thyroid follicular cell proliferation.

### Analysis of the Glis3 -Pax8Cre thyroid gland transcriptome

**Figure 4:**
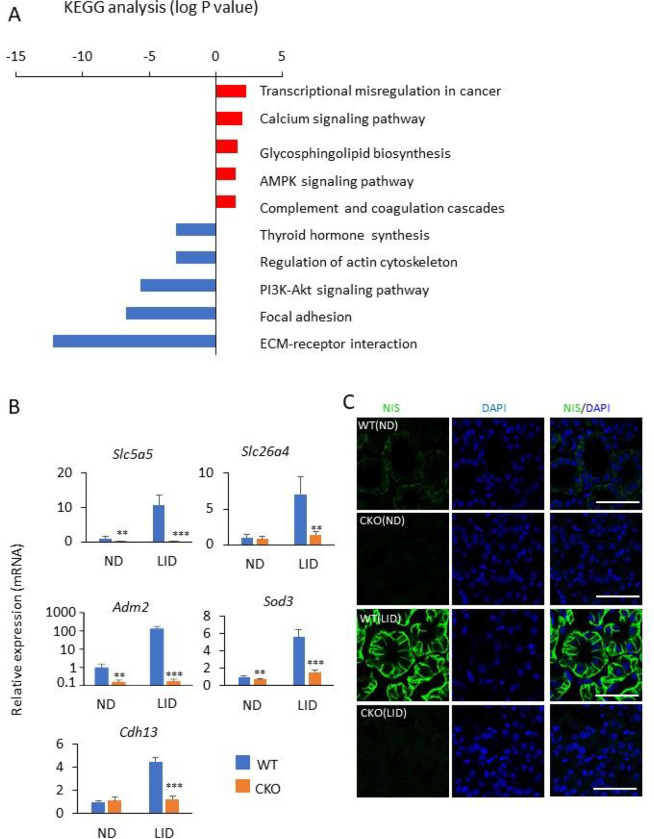
Transcriptome analysis of thyroid glands from female WT(LID) and *Glis3*-Pax8Cre(LID) mice. **A** KEGG pathway analysis of genes up-regulated (red) and down-regulated (blue) in *Glis3*-Pax8Cre(LID) thyroid glands compared to those of WT(LID). **B** Comparison of the expression of several TSH-inducible genes in thyroid glands from WT(ND), WT(LID), *Glis3*-Pax8Cre(ND), and *Glis3*-Pax8Cre(LID) mice by QRT-PCR analysis. ** p<0.01, *** p<0.001. **C** The expression of SLC5A5 (NIS, green) was examined in thyroid glands from WT(ND), WT(LID), *Glis3*-Pax8Cre(ND), and *Glis3*-Pax8Cre(LID) mice by immunofluorescence staining. DAPI (blue). Scale bars: 50 mm.

**Figure 5:**
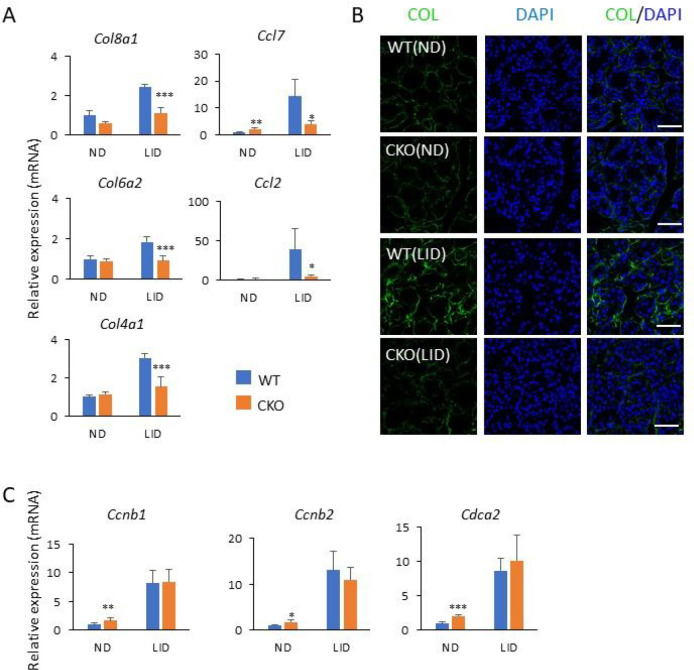
The expression of ECM- and inflammation-related genes, but not those of cell cycle-related genes, was suppressed in thyroid glands from *Glis3*-Pax8Cre(LID) mice. **A** QRT-PCR analysis of the expression of several collagen and inflammatory genes in thyroid glands from WT(ND), WT(LID), *Glis3*-Pax8Cre(ND), and *Glis3*-Pax8Cre(LID) mice. **B** Collagen expression in the thyroid glands was examined by immunofluorescence staining with a pan-collagen antibody (COL; green). DAPI (blue). Scale bars: 50 mm. **C** QRT-PCR analysis of several cell cycle-related genes. The expression of cell cycle-related genes was not repressed in *Glis3*-Pax8Cre(LID) consistent with data in Figure 3. * p<0.05, ** p<0.01, *** p<0.001. In contrast to ubiquitous Glis3-knockout mice [33], cell proliferation-related genes were not suppressed in *Glis3*-Pax8Cre(LID) mice.

To obtain insights into the differences in gene expression between thyroid glands from WT and *Glis3*-Pax8Cre mice, we performed RNA-Seq analysis (Supplementary Table 2). KEGG analysis of genes down-regulated in *Glis3*-Pax8Cre(LID) thyroids compared to those of WT(LID) (fold change > 2; FDR < 0.01), identified ECM-receptor interaction, focal adhesion, and thyroid hormone synthesis among the top pathways, while analysis of up-regulated genes identified transcriptional-misregulation-in-cancer, calcium and AMPK signaling among the top pathways (Fig. 4A and Supplementary Table 3). The expression of several thyroid hormone biosynthetic and TSH-induced genes, including *Slc5a5*, *Slc26a4*, *Adm2*, *Sod3*, *Cdh13*, *Duox2*, and *Duoxa2* that were greatly induced in WT(LID) compared to WT(ND), were significantly repressed in thyroids from *Glis3*-Pax8Cre(LID) mice (Table 1 and Fig. 4B), consistent with our previous study of ubiquitous *Glis3*KO mice [33]. NIS protein expression was dramatically increased in WT(LID), but not in *Glis3*-Pax8Cre(LID) thyroids (Fig. 4C). Similarly, the increase in the expression of several ECM-related and inflammatory genes, including *Col18a1*, *Col6a2*, *Col4a1*, *Ccl7*, *Ccl2*, and *Itga2*, observed in WT(LID) thyroids was significantly suppressed in *Glis3*-Pax8Cre(LID) thyroid glands (Table 1 and Fig. 5A). The increase in collagen mRNA expression in WT(LID) and its suppression in *Glis3*-Pax8Cre(LID) thyroids correlated with the level of immunofluorescent staining for pan-collagen (Fig. 5B).

Most importantly and in contrast to ubiquitous *Glis3*KO(LID) mice [33], the expression of cell cycle regulatory genes, including *Ccnb1*, *Ccnb2*, and *Cdca2*, was not suppressed in *Glis3*-Pax8Cre(LID) thyroid glands (Table 1; Fig. 5C) consistent with our data showing little difference in the percentage of PAX8^+^EdU^+^ cells (Fig. 3D). These data supported the concept that the inhibition of thyroid follicular cell proliferation in ubiquitous *Glis3*KO mice appears to be related to changes in gene expression in other cell types that subsequently affect the proliferation of these cells. Together, these observations indicate that in contrast to TH biosynthetic genes, which transcription is directly regulated by GLIS3, GLIS3 does not play a major role in the direct transcriptional regulation of cell proliferation-regulatory genes in thyroid follicular cells in mice fed a LID. These findings are consistent with the concept that TSH regulates proliferation and TH-biosynthesis in thyroid follicular cells through activation of different signaling pathways [7].

Analysis of thyroid TF expression showed that *Pax8* expression, but not that of *Nkx2*.*1* and *Foxe1*, was increased in both *Glis3*-Pax8Cre(ND) and *Glis3*-Pax8Cre(LID) mice compared to that of WT(ND) and WT(LID) (Supplementary Fig. S4). However, the expression of *Pax8*, *Nkx2.1*, and *Foxe1* was not significantly different between thyroids from *Glis3*-Pax8Cre(ND) and *Glis3*-Pax8Cre(LID) mice indicating that the suppression of gene expression in *Glis3*-Pax8Cre(LID) thyroid is independent of the changes in the expression of these thyroid TFs.

The transcriptome and QRT-PCR analyses were carried out with thyroid glands from female mice, analysis of the expression of several genes, including cell cycle, ECM, inflammation, and TF genes in thyroids from male mice showed a very similar pattern as that of female mice (Supplementary Fig. 5).

### Correlation between GLIS3 protein and TSH levels

**Figure 6:**
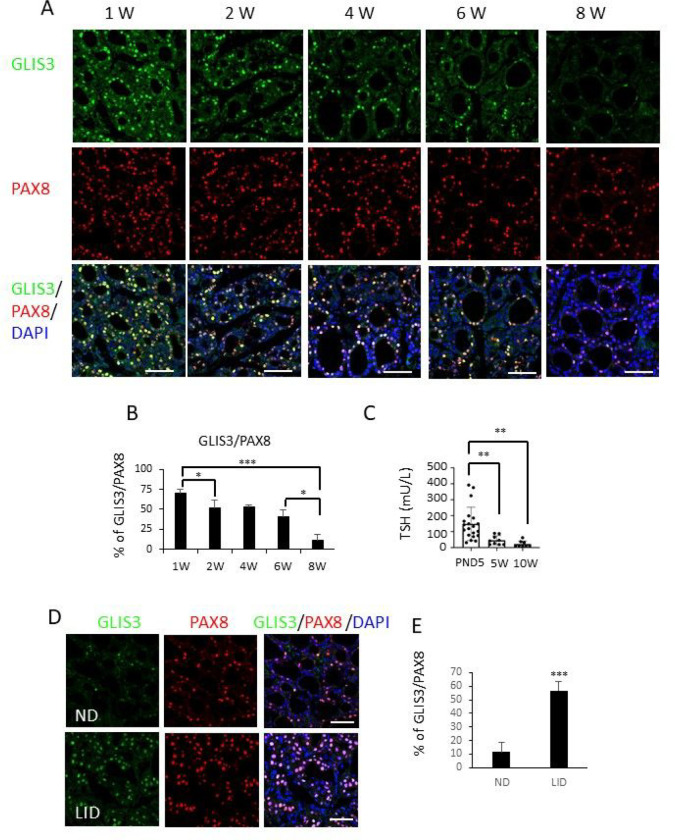
GLIS3 protein expression in thyroid follicular cells correlated with serum TSH levels. **A, B** GLIS3 protein expression is greatly decreased during early postnatal development. Expression of endogenous GLIS3 and PAX8 protein was examined in thyroid glands from 1-, 2-, 4-, 6-, and 8-week-old Glis3-EGFP mice by immunofluorescence staining with GFP (green) and PAX8 (red) antibodies. Nuclei were stained with DAPI (**A**). The percentage of PAX8^+^ cells that were GLIS3^+^ (from **A**) was calculated and plotted (**B**). Scale bars: 50 mm. **C** Serum TSH levels in WT mice were examined at PND5, 5- and 10-weeks. ** p<0.01. **D, E** GLIS3 protein expression in thyroid follicular cells is increased in mice fed a LID (high serum TSH). 8-week-old *Glis3*-EGFP mice were fed an ND and LID for 2 weeks before the expression of GLIS3 and PAX8 was examined in thyroid glands by immunofluorescence staining (**D**). The percentage of PAX8^+^ cells (from **D**) that are GLIS3^+^ was calculated and plotted (**E**). Scale bars: 50 mm.

Our studies demonstrated that GLIS3 plays a critical role particularly in the transcriptional regulation of several thyroid hormone biosynthetic and TSH-inducible genes under conditions when TSH levels are elevated, such as in LID (Table 1 and Fig. 4B)[33, 60]. This led to the hypothesis that GLIS3 protein levels and/or activity itself might be controlled by a TSH/TSHR-dependent signaling pathway. This concept was supported by observations showing a correlation between the decrease in GLIS3 and TSH levels during the first 2 postnatal months. Both the intensity of GLIS3 protein staining in follicular cells and the percentage of PAX8^+^GLIS3^+^ cells in *Glis3*-EGFP mice steadily decreased during this period (Fig. 6A, B) and was accompanied with a similar decline in postnatal blood TSH levels (Fig. 6C). The correlation between GLIS3 protein and TSH levels was strengthened by observations showing that GLIS3 staining and the percentage of PAX8^+^GLIS3^+^ cells were greatly increased in *Glis3*-EGFP mice fed a LID, a condition in which blood TSH level is greatly elevated (Fig. 6D, E). These changes in GLIS3 protein did not strongly correlate with alterations in Glis3 mRNA expression (Supplementary Fig. 6) suggesting that the higher levels of GLIS3 protein expression under conditions of elevated TSH, might be due to an increase in protein stability or rate of translation rather than increased transcription. The increase in GLIS3 protein might be part of the mechanism by which GLIS3 induces the transcriptional activation of target genes, such as *Slc5a5*, in thyroid follicular cells of LID mice.

### Link between PKA and GLIS3 transcriptional activity

**Figure 7:**
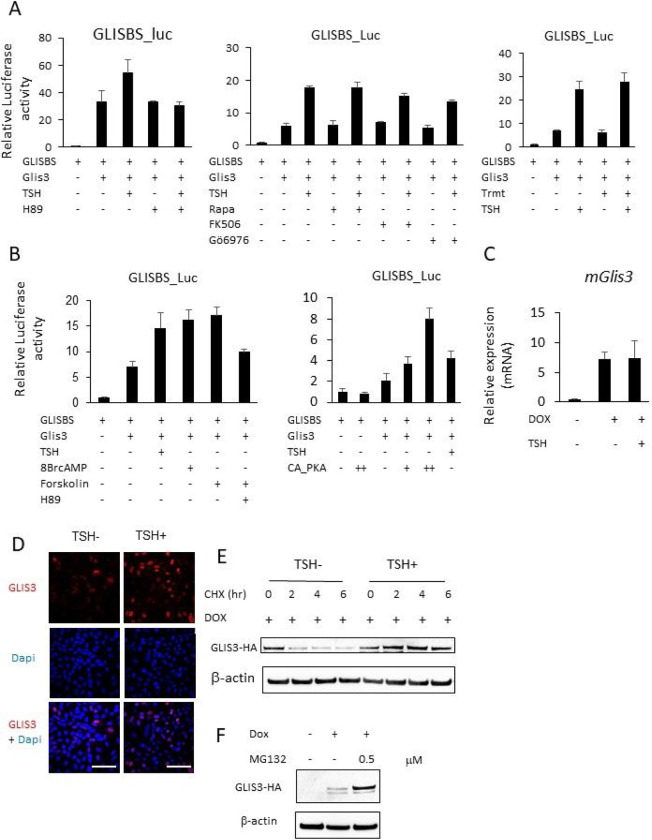
TSH and PKA agonists enhanced GLIS3-mediated transcriptional activation. **A** GLISBS-dependent transcriptional activation of the Luc reporter by GLIS3 was examined in PCCl3 cells as described in Materials and Methods. Addition of TSH stimulated GLIS3-mediated transcriptional activation. This increase was inhibited by the PKA inhibitor H89, whereas treatment with the mTOR inhibitor rapamycin (Rapa), the calcium signaling inhibitor FK506, the PKC inhibitor Gö6976, or the ERK inhibitor Trametinib (Trmt) had little effect on the TSH-stimulated transcriptional activation by GLIS3. **B** Addition of 8BrcAMP or the adenylyl cyclase activator forskolin enhanced transcriptional activation by GLIS3. The increase by forskolin was inhibited by the PKA inhibitor H89. Expression of constitutively active PKA (CA_PKA) enhanced GLISBS-dependent transcriptional activation of the Luc reporter by GLIS3. **C, D** TSH enhanced GLIS3-HA protein expression in Dox-treated PCCl3-pIND20-Glis3 cells without increasing Glis3 mRNA. GLIS3-HA was induced by Dox in PCCl3-pIND20-Glis3 cells, cultured in the absence of TSH for 4 days, and then treated with or without TSH for 24 h before Glis3-HA mRNA and GLIS3-HA protein were examined by QRT-PCR analysis (C) and confocal microscopy (D), respectively. Scale bars: 50 mm. **E** The PCCl3-pIND20-Glis3 cells were treated with Dox for 72 h and subsequently treated with 10 mg/ml cycloheximide in the presence or absence of TSH. Cells were harvested at the indicated time, nuclear extract prepared, and GLIS3-HA levels examined by Western blot analysis. **F** PCCl3-pIND20-Glis3 cells were treated with Dox for 72 h and subsequently with 0.5 mM of MG132 for 5 h before cells were harvested at the times indicated, and nuclear extracts examined for GLIS3-HA expression by Western blot analysis.

To obtain further support for the hypothesis that GLIS3 activity is regulated by TSH signaling, we examined the effect of TSH on GLIS3-mediated transcriptional activation of a GLISBS-dependent luciferase reporter in rat thyrocyte PCCl3 cells. Addition of TSH increased GLIS3-mediated transcriptional activation of the reporter 2- to 3-fold (Fig. 7A, B) without causing a change in the level of Glis3 mRNA expression (Fig. 7C). In the absence of GLIS3, TSH did not increase GLISBS-dependent activation (data not shown). It is well-established that interaction of TSH with TSHR induces the activation of several protein kinases, including PKA, phospholipase C (PLC), PI3K, mTOR, ERK, and Ca^++^-mediated signaling [7, 52, 61, 62]. To examine whether any of these downstream kinase pathways are involved in the regulation of GLIS3 activity, we analyzed the effect of several kinase inhibitors on GLIS3-mediated transcriptional activation. Addition of the PKA inhibitor H89 significantly suppressed the increase in GLIS3-mediated transactivation by TSH (Fig. 7A), whereas inhibition of the mTOR, Ca^++^ or ERK pathways by, respectively, rapamycin, FK506 or trametinib, had little effect, while transactivation was slightly reduced by the PKC inhibitor Gö6976 (Fig. 7A). A role for PKA activation in the regulation of GLIS3-mediated transcriptional activation by TSH was further supported by data showing that co-expression of a constitutively active form of PKA and treatment with 8BrcAMP or the adenylyl cyclase agonist, forskolin, enhanced GLIS3-dependent transcriptional activation and that this increase was significantly inhibited by H89 (Fig. 7B). Together, these results are consistent with the concept that activation of PKA is part of the mechanism by which by TSH enhances GLIS3 transcriptional activation of target genes.

**Figure 8:**
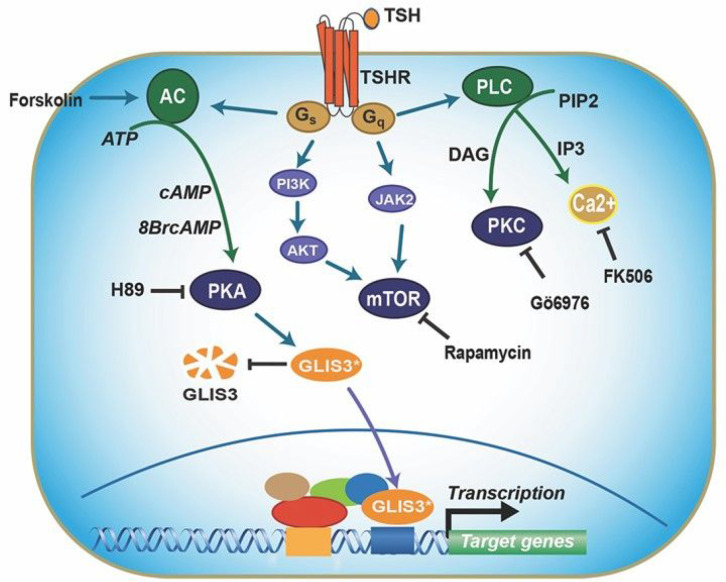
Schematic showing a model of the mechanistic link between TSH signaling and its regulation of GLIS3 transcriptional activity. Binding of TSH to TSHR leads to activation of several protein kinase pathways and increased GLIS3 protein stability and transcriptional activity. TSH, the PKA activator 8BrcAMP, the adenylyl cyclase activator forskolin, and expression of constitutively active PKA enhance GLIS3 transcriptional activity at least in part by stabilizing GLIS3 protein. The PKA antagonist H89 suppressed the TSH-mediated increase, whereas inhibition of mTOR, PKC, Ca^++^ or ERK pathways by, respectively, rapamycin, Gö6976, FK506 or trametinib, had little effect. The stimulation in GLIS3 transcriptional activity by the TSH-TSHR-PKA pathway appears to be at least in part due to increased GLIS3 protein stability. This mechanism of regulation provides an explanation for the dramatic increase in GLIS3 protein expression and the subsequent induction of GLIS3 target genes, including several thyroid hormone biosynthetic genes, in thyroid follicular cells of mice fed a LID.

Since TSH treatment did not significantly change Glis3 mRNA levels (Fig. 7C), one possible mechanism by which TSH enhances GLIS3 transcriptional activity is increasing GLIS3 protein stability. To study this, we examined the effect of TSH on GLIS3 protein in PCCl3 cells expressing doxycycline (Dox)-inducible GLIS3-HA (PCCl3-pIND20-Glis3) and examined GLIS3 protein expression by immunofluorescence staining and Western blot analysis. These analyses showed that addition of TSH significantly increased GLIS3 protein expression in Dox-treated PCCl3-pIND20-Glis3 cells without changing Glis3 mRNA expression (Fig. 7C–E). We further showed that treatment with the proteasome inhibitor MG132 enhanced GLIS3 protein stability (Fig. 7F). Together, these data are consistent with the hypothesis that the stimulation of GLIS3 transcriptional activity by TSH is at least in part mediated via a PKA-dependent increase in GLIS3 protein stability (Fig. 8).

## Discussion

Loss of GLIS3 function in both humans and mice causes CH [15, 16, 18, 33]. However, whether this is related to thyroid dysgenesis or dyshormonogenesis has not been clearly established. Analysis of GLIS3 protein expression during mouse thyroid development demonstrated that GLIS3 protein was first detectable in thyroid follicular cells at E15.5, a time during which thyroid follicles are being formed (Fig. 1 and Supplementary Fig. 1) [4, 8]. We further show that thyroid gland morphology and the formation of thyroid follicles are not greatly altered in E17.5 and neonatal *Glis3*KO mice (Fig. 2A, Supplementary Fig. 2). These data indicate that GLIS3 is not required for early thyroid development in mice and that the development of CH in *Glis3*KO mice is due to dyshormonogenesis rather than thyroid dysgenesis. This contrasts the role of *glis3* in zebrafish, in which *glis3* has been shown to play an important role in thyroid development [63]. Moreover, unlike *glis3* knockdown in zebrafish, the expression of NKX2.1 and PAX8 was not impaired in thyroid gland in *Glis3*KO mice (Fig. 2B and C). Whether the development of CH in human patients with GLIS3-de ciency is related to thyroid dysgenesis or dyshormonogenesis has been inconclusive and shown to vary among patients [16, 18, 20–22, 24, 25, 29, 64, 65]. This variability might be attributed to the highly oligogenic nature of CH [27, 29, 32]. Our study further shows that the onset of GLIS3 expression is distinct from that of the thyroid TFs, PAX8, NKX2.1, FOXE1, and HHEX, which are critical for early thyroid gland development and which loss of function causes thyroid dysgenesis or athyreosis [3, 4, 6, 9, 10, 12, 66]. Several of these TFs also play a role postnatally in the regulation of several thyroid functions, including TH biosynthesis. We recently reported that PAX8 and NKX2.1 are bound to regulatory regions of the *Glis3* gene, which would be consistent with the concept that these factors have a role in the transcriptional activation of *Glis3* during thyroid development [33, 60]. Interestingly, the time interval at which *Glis3* expression is induced parallels that of a major GLIS3 target gene, *Slc5a5*, which expression is induced during E15.5 and 16.5 and greatly repressed in E17.5 *Glis3*KO thyroids (Fig. 1B and 2F). Impairment in iodide transport in NIS knockout mice was reported to cause severe hypothyroidism [67]. Together, these findings are consistent with our conclusion that CH in *Glis3*KO mice is due to dyshormonogenesis.

We previously demonstrated that under LID conditions not only the activation of thyroid hormone biosynthetic genes was suppressed in ubiquitous *Glis3*-deficient mice, but also the induction of thyroid follicular cell proliferation, the expression of cell cycle genes, and the activation of the mTOR pathway [15, 33]. We also reported that, in addition to congenital hypothyroidism, these mice develop neonatal diabetes and hypoinsulinemia [39, 58]. IGF-1 and insulin, together with TSH, have been reported to play a critical role in the regulation of gene expression and proliferation in thyroid follicular cells [51–56] raising the possibility that the pancreatic phenotype, including hypoinsulinemia, and conceivably changes in other tissues in ubiquitous *Glis3*-deficient mice might influence the function and gene expression in thyroid follicular cells. This hypothesis was supported by observations showing that in contrast to ubiquitous *Glis3*-KO(LID) mice, thyroid follicular cell proliferation, expression of cell cycle genes, including *Ccnb1*, *Ccnb2*, and *Cdca2*, and activation of the mTOR, a pathway that promotes cell proliferation, were not repressed in *Glis3*-Pax8Cre(LID) mice but induced to a similar degree as in WT(LID) mice (Fig. 3C–E and Fig. 5C). We further observed that proliferation of thyroid follicular cells (EdU^+^ cells) and the expression of the cell cycle genes were slightly higher in *Glis3*-Pax8Cre(ND) mice than in WT(ND) mice. This might be due to the 3-fold higher level of TSH in *Glis3*-Pax8Cre(ND) mice compared to WT(ND) mice (Fig. 3B). These data indicate that GLIS3 does not play a major role in the regulation of thyroid follicular cell proliferation and suggest the suppression of thyroid follicular cell proliferation in ubiquitous *Glis3*-deficient mice does not involve direct transcriptional regulation of cell cycle-related genes by GLIS3, but is related to abnormalities in other tissues, such as hypoinsulinemia that indirectly affect thyroid follicular cell proliferation. This conclusion is consistent with our cistrome analysis showing that GLIS3 binds to very few cell cycle-related genes [60]. In contrast to cell proliferation-regulatory genes, the expression of several TH-inducible genes, including *Slc5a5*, *Slc26a4*, *Adm2*, *Sod3*, and *Cdh13*, remain dramatically suppressed in *Glis3*-Pax8Cre(LID) thyroid as we observed in ubiquitous Glis3-deficient mice, consistent with the conclusion that their transcription is directly regulated by GLIS3 [60]. In addition, these findings support the concept that TSH regulates proliferation and TH-biosynthesis in thyroid follicular cells through different protein kinase signaling pathways [7].

The largest repression of GLIS3 target genes was observed in thyroids from *Glis3*KO and *Glis3*-Pax8Cre mice under LID conditions when TSH levels are highly elevated (Fig. 3B)[33]. The induction of the transcriptional activation of GLIS3 target genes in thyroid follicular cells in mice fed a LID coincides with the significant increase in GLIS3 protein expression (Fig. 6). These observations indicated a possible link between the TSH/TSHR signaling pathway, the regulation of GLIS3 protein, and the activation of GLIS3 target genes. This was supported by data showing that TSH enhanced GLIS3-mediated transcriptional activation and GLIS3 protein expression in PCCl3 cells (Fig. 7A, D). This induction was not due to a change in the level of Glis3 mRNA expression (Fig. 7C) suggesting that it might be due to increased GLIS3 protein stability or rate of translation. We provided evidence indicating that the stimulation of GLIS3 transcriptional activity is at least in part due to increased GLIS3 protein stability (Fig. 7E, F). GLIS3 protein stability and GLIS3-dependent transcriptional activation of target genes might be controlled by posttranslational modification(s) of GLIS3 that are mediated by TSH/TSHR-induced activation of (a) downstream kinase pathway(s) [11, 52, 59, 68]. Study of the effect of several kinase inhibitors on TSH-induced stimulation of GLIS3 transcriptional activity revealed that the PKA antagonist H89 suppressed this increase, but that inhibition of several other kinase pathways (ERK, PKC, mTOR, Ca2^+^) had relatively little effect. A role for PKA in mediating the effect of TSH was supported by data showing that the PKA agonist 8BrcAMP and the adenylyl cyclase agonist, forskolin, similarly enhanced GLIS3-mediated transcriptional activation and that this stimulation was inhibited by H89. A role for PKA was further strengthened by observations demonstrating that expression of a constitutively active PKA stimulated GLIS3-mediated transcriptional activation (Fig. 7B). Further studies are needed to identify the amino acid(s) within GLIS3 that are phosphorylated by PKA and are critical for regulating GLIS3 protein stability and activity. Together, our study reveals a link between TSH signaling and its regulation of GLIS3 protein activity and transcriptional activation GLIS3 target genes, including several TH biosynthesis-related genes. We further provide evidence for a role of the PKA signaling pathway in mediating the transcriptional regulation of several TSH-induced genes by GLIS3 in thyroid follicular cells (Fig. 8). Strategies to regulate GLIS3 expression and/or its transcription activity might provide new therapeutic approaches in the management of hypo- and hyperthyroidism.

## Data Availability

RNA-Seq data were deposited in the NCBI Gene Expression Omnibus (GEO) database https://www.ncbi.nlm.nih.gov/geo/ under accession GSE207775.

## Abbreviations

GLIS3: Gli-similar 3
CH: congenital hypothyroidism
TH: thyroid hormone
GLISBS: GLIS binding site
TF: transcriptional factor
TSH: thyroid stimulating hormone
TSHR: thyroid stimulating hormone receptor
ECM: extracellular matrix
NDH: neonatal diabetes and congenital hypothyroidism
PND: postnatal day
KO: knockout
CKO: conditional knockout
PKA: protein kinase A
ND: normal diet
LID: low iodine diet
NIS: sodium-iodide symporter
DUOX2: dual oxidase 2
FOXE1: forkhead box E1
HHEX: hematopoietically expressed homeobox
NKX2.1: NK2 homeobox 1
PAX8: paired box 8
PDS: pendrin
TPO: thyroid peroxidase
TG: thyroglobulin
cAMP: cyclic AMP
ChIP: Chromatin immunoprecipitation
EGFP: enhanced green fluorescent protein
